# The Contribution of Social and Structural Determinants of Health Deficits to Mental and Behavioral Health Among a Diverse Group of Young People

**DOI:** 10.3390/ijerph22071013

**Published:** 2025-06-26

**Authors:** Kimberly J. Mitchell, Victoria Banyard, Deirdre Colburn

**Affiliations:** 1 Crimes Against Children Research Center, University of New Hampshire, Durham, NH 03824, USA; deirdre.colburn@unh.edu; 2 New Brunswick School of Social Work, Rutgers University, New Brunswick, NJ 08901, USA; victoria.banyard@rutgers.edu

**Keywords:** suicide, drug overdose, social determinants of health, marginalized identities, latent class analysis, health disparities

## Abstract

A growing knowledge base highlights the importance of accounting for a variety of social and structural determinants of health (SDOH) when understanding mental and behavioral health among adolescents and young adults. The objective of the current study is to examine patterns of self-reported SDOH deficits and characterize participant health indicators and social identity across classes. Data is from a cross-sectional national study of young people who were recruited through study advertisements on social media and surveyed online. Data were collected between June 2022 and October 2023. Eligibility included (1) ages 13–22 years, (2) living in the United States, and (3) proficient in English. Health indicators included suicide attempts, suicidal ideation, drug overdose, perceived likelihood of living to age 35, non-suicidal self-injury, recent alcohol use, and depression. Five classes of SDOH deficits were identified: (1) Economic Instability, (2) Low Overall SDOH Deficits, (3) High Social SDOH Deficits (adversity and discrimination), (4) High Economic SDOH Deficits, and (5) High Overall SDOH Deficits. Differences across class by health indicators and marginalized identity were found, with high proportions of gender minority and sexual minority youth in both the High Overall SDOH Deficit group and the High Social SDOH Deficit classes. Black youth were more likely to be part of the High Economic SDOH Deficits class. The findings encourage a public health approach that recognizes that improving the health of today’s young people must be connected to policies that reduce poverty, improve neighborhoods, and increase access to basic goods, services, and healthcare.

## 1. Introduction

There is currently a mental health crisis for adolescents and young adults. Suicide is one of the most significant mental health risks that young people face. In 2021, suicide was the second and third leading cause of death among 10–14 and 15–24-year-olds in the United States, respectively [1]. Other indicators of mental health include symptoms of depression and engaging in non-suicidal self-injury behaviors. These problems also occur at alarming rates [2,3,4]. Additionally, between 2020 and 2021, drug overdose deaths in these age groups increased 14%, to 32.4 per 100,000 people. Orientation to the future, such as perceived likelihood of living to age 35, is also an important health indicator, as low perceptions are linked to risky health behaviors and negative outcomes [5,6]. As these mental health indicators often co-occur [7,8], there may be shared explanatory factors that can be examined together to provide a fuller picture of youths’ experiences, although to date they are most often studied separately [9]. We need to better understand these interconnections and how they vary based on social locations to create culturally responsive prevention and response strategies.

Rates of suicidal thoughts and behaviors are not evenly distributed; youth who identify with identities that experience health disparities (sexuality, gender, race) often report higher rates of suicide attempts [10,11]. Rates of death by suicide are elevated among sexual and gender minority youth compared to their non-sexual- and non-gender-minority peers [3,4,5,6] and higher among Black and Latino youth [11]. Increased risk of substance use disorders among adolescents with marginalized identities are also noted [2]. For example, research consistently finds higher rates of substance misuse among sexual and gender minority youth compared to their non-sexual- and non-gender-minority peers [3,4,5,6]. Recent research further suggests that rates of overdose are also unequal by race [12,13,14].

Minority Stress Theory (MST) [15] has been used to understand why young people from marginalized groups (based on gender, sexuality, race, ethnicity) are at higher risk for suicide. Discrimination and victimization contributes to a greater burden of adversity and internalized stress for these young people compared to their cisgender, heterosexual, or White peers [11,16,17,18,19]. MST is linked to suicide attempts specifically using theories like the Three-Step Theory (3ST) [20] that states that suicide begins with conditions that create pain and hopelessness, which leads to disconnection, and when combined, enhances the capacity to engage in suicidal behaviors. The distal factors of victimization and discrimination described in MST create trauma that may contribute to the pain and hopelessness and also disconnection from others reflected in steps 1 and 2 of 3ST [21]. MST has also been used to understand substance misuse [22,23], rates of depression and anxiety [24], and non-suicidal self-injury [17,25].

Social Determinants of Health (SDOH) theory [26,27,28,29] is now used widely to understand how the conditions people and groups live in produce different health concerns and health outcomes due to systemic and contextual factors. While measures of social factors like discrimination and victimization are included in SDOH research [30], SDOH covers a wider range of ecological factors [31]. These are captured in domains including economic (e.g., income, food security), social and community (e.g., discrimination, adversity), built environment (e.g., physical disrepair of housing or neighborhood), and health systems (e.g., access to dentist or mental health provider) [18,19,20,21,32]. These external, social, and structural variables are beyond an individual’s control but may result in health disparities because of systemic patterns of exclusion from resources. Indeed, people who have more SDOH deficits have poorer mental and behavioral health indicators [10,21,22,23,24]. A recent study found links between SDOH (especially food insecurity and loneliness) and depression; gender was a significant moderator [33]. A recent review highlights the need to study SDOH among sexual and gender minority communities [34] and among racially diverse young people [35]. Studies also show links between economic and health-related SDOH and suicide ideation [36] among young people. A review of meta-analyses across the lifespan found significant SDOH risk for suicide attempts, including experiences of victimization and bias bullying (a social domain), insecure housing, and natural disasters (structural/built environment) [37]. As such, SDOH theory is an important model for identifying potentially modifiable factors in individuals’ or groups’ social, physical, economic, and health contexts that may explain these disparities. These factors, in turn, can be a focus of prevention and intervention efforts to create health equity [32].

There are limitations in our current understanding of links between SDOH and suicide and other health indicators among adolescents and young adults. Studies of minority stress focus on discrimination and the internalized coping processes that result in variables in the social domain of SDOH only. SDOH overall has been more commonly used to study physical health or disease disparities than mental health outcomes [38,39,40,41]. The current study builds upon extant research that documents the importance of considering social, economic, built environment, and health access as determinants of health in suicide attempts, drug overdoses, and other health indicators. We assess patterns of SDOH deficits using latent class analysis and compare how participants’ health indicators and marginalized identities characterize the classes.

## 2. Materials and Methods

### 2.1. Participants and Recruitment

Project Lift Up is a national longitudinal study designed to understand exposure to self-directed violence in social networks. A cohort of 4981 adolescents and young adults aged 13–22 years was recruited between 13 June 2022 and 30 October 2023 (see Table 1 for sample characteristics). Baseline data was used for the current analyses. The protocol was reviewed and approved by the University of New Hampshire Institutional Review Board. A waiver of caregiver permission was granted for minors because requiring caregiver consent could potentially place youth in situations where their sexual experiences and/or sexual attraction could be unintentionally disclosed to their parents. Appropriate mechanisms were in place to protect children, including help resources provided to all participants and referrals to a team clinician for additional telehealth outreach if a participant reported a mental health problem and was not currently in treatment.

Participants were recruited through study advertisements on social media, encouraging youth to ‘have their voice heard’ and ‘make a difference.’ Survey aims were not mentioned, to help reduce self-selection bias. Those who clicked on the advertisement were linked to a secure survey website. The first page provided a study description and screening questions to determine eligibility (13–22 years of age, living in the United States, and proficient in English). Eligible participants then read an assent/consent form and indicated their willingness to participate before continuing with the main survey. The American Association for Public Opinion Research (AAPOR) cautions against reporting traditional response rates for online non-probability samples [26]. The participation rate of this study, defined by AAPOR as the number of usable responses (i.e., complete main survey responses) divided by the number of initial invitations requesting participation (i.e., eligible screener responses), was 21.0%.

Participants were given a $15 Amazon gift code incentive for completing the survey. Ineligible youth were directed to a web page that included links to general resources for youth (e.g., https://youngwomenshealth.org, accessed on 10 June 2025). To promote a diverse sample, demographic quotas were identified based on the intersection of age (13–17 vs. 18–22), sex assigned at birth, sexual or gender minority identify, and race or ethnic minority identity. Once the targeted number of participants in a particular group had been achieved (e.g., aged 13–17, cisgender girls), subsequent participants in this group were ineligible. A robust series of data quality protocols were used to identify untrustworthy data, including age verification, reverse lookup of IP addresses and phone numbers, review of names, addresses, social media accounts, time to survey completion, attention check questions, and direct participant outreach, as needed.

### 2.2. Measures

#### 2.2.1. Health Indicators

Suicide attempt. This item is from the DSM-5-TR Self-Rated Level 1 Cross-Cutting Symptom Measures for Ages 11–17: [42] Participants were asked, “Have you ever tried to die by suicide?” Response options were yes, no, prefer not to answer.

Suicidal ideation. This item is from the DSM-5-TR Self-Rated Level 1 Cross-Cutting Symptom Measures for Ages 11–17: [42] “In the past two weeks, have you thought about killing yourself or dying by suicide?” Response options were yes, no, prefer not to answer.

NSSI. Participants were prompted, “Thinking of things you may have done to yourself on purpose, have you ever…” (a) cut or carved your skin, (b) hit yourself, (c) pulled your hair, (d) burned your skin, and (e) some other type of self-injury [43]? For each endorsed form of NNSI, follow-up questions asked how many times the participant had performed this in the past 12 months. Any NSSI lifetime and past year variables were created for analyses.

Drug overdose. Participants were asked, “Have you ever overdosed on medication, pills, or other drugs so that you got really sick and had to go to the hospital?” (yes/no/prefer not to answer). This item was modified from an existing non-victimization life adversity measure [44] to reflect one’s own overdose instead of exposure to someone else’s.

Alcohol use. This item is from the DSM-5-TR Self-Rated Level 1 Cross-Cutting Symptom Measures for Ages 11–17: [42] Participants were asked, “During the past two weeks, have you had an alcohol beverage (beer, wine, liquor, etc.)?” Response options were yes, no, prefer not to answer.

Perceived percent chance of living to age 35. Participants were asked what they think the chances were that they would live to age 35. Response options ranged from 0 to 100% [10].

Depression and Anxiety. The Revised Children’s Anxiety and Depression Scale (RCADS) [45,46] is a self-report, 25-item questionnaire that assesses symptoms of depression and anxiety in children and adolescents. It captures symptoms related to major depressive disorder (10 items), panic disorder (3 items), social phobia (3 items), generalized anxiety disorder (3 items), separation anxiety disorder (3 items), and obsessive–compulsive disorder (3 items). For the purposes of the current analysis, all items were combined to generate a total internalizing score.

#### 2.2.2. Social and Structural Determinants of Health Deficits

SDOH screening tools exist but are designed primarily for the in-person assessment of adults in clinical settings. Items and scales used in the current study were drawn from this work [47] and then modified and piloted with youth. Items cover four domains of SDOH deficits, detailed below.

Economic instability was captured with three items (each on 5-point scale ranging from 0 (never) to 4 (always)): “How often did this describe you or your family in the past 12 months? We did not have enough money to pay the bills.”; “In the past 12 months, how often has your cell phone been turned off because you or your family did not have enough money to pay the bill?”; and “In the past 30 days, how often did you skip meals or eat less because you or your family didn’t have enough money for food?” One additional item asked participants to describe their family’s income with the following response options: lower than the average family, about the same as the average family, or higher than the average family.

Social context

*Lifetime non-victimization adversity* due to non-violent traumatic events and chronic stressors was measured using 10 yes/no items [44] (for example, serious illnesses, accidents, family homelessness). Items were summed to reflect a total adversity count.

*Discrimination* was measured using the Everyday Discrimination Scale [48] and asked participants, “In your day-to-day life, how often do any of the following things happen to you?” Response options ranged from 0 (never) to 5 (almost every day). Sample items included being treated with less courtesy and respect than others and being threatened or harassed.

Health care

*Barriers to access to mental health care*. Participants were asked to rate each of five possible concerns that might affect their decision to see treatment for a mental health problem from a professional, like a psychologist or counselor, including not knowing where to get help, not having a way to get to a treatment center, too hard to schedule an appointment, difficulty getting time off work or school for treatment, and cost of treatment. Response options ranged from 1 (strongly disagree) to 5 (strongly agree). Items were summed to create a total barriers score.

*Dental visit*. Participants were asked, “When was the last time you saw a dentist for a check-up, exam, teeth cleaning, or other dental work?” (in the past 12 months, between 1 and 2 years ago, more than 2 years ago, I have never been to the dentist). This item was recoded to reflect more than 2 years (or never) vs. more recently.

Neighborhood and built environment

*Poor home conditions*. Eight items covered problems where the participant currently lived [47]. Participants were told to think about their permanent place of residence, not a dorm room or other temporary housing. The items included bugs everywhere, mold, lead paint or lead pipes, not enough heat, the oven or stove does not work, there are no smoke detectors, or they do not work, water leaks, no or frequent loss of electricity. Response options for each were yes/no and summed to create a total.

*Neighborhood disorder*. Twelve items measured perceptions of the severity of different neighborhood conditions [49] and were edited to be more meaningful to youth. Participants were asked to rate each of the following as to whether it was no problem, a minor problem, or a serious problem in their neighborhood (“by neighborhood we mean the street you live on and a few streets around it”): gangs, graffiti, drugs, homelessness, buildings with broken windows or other damage, abandoned or boarded-up buildings, violent crime, police or ambulance sirens at night, gun shots at night, fighting, trash or litter, break-ins or burglaries.

We created z-scores for each SDOH measure to ensure they had relatively similar scores within each group. The dental visit variable was an exception; this was included as the dichotomous coded variable described above. Supplemental Table S1 provides descriptive statistics of the original and transformed items. Supplemental Table S2 provides pairwise correlations among each of these SDOH transformed items.

#### 2.2.3. Demographic Characteristics

Sexual and gender identity. To measure sexual identity, participants were asked which best represents how they thought of themselves: gay; lesbian, bisexual; heterosexual; queer; polysexual, omnisexual, sapiosexual, or pansexual; demisexual; asexual; two-spirit; do not think of myself as having sexuality; do not use labels to identify myself; questions/I am unsure, something else. These multiple response options were reduced to single categories for analyses: (1) exclusively heterosexual; (2) gay or lesbian, (3) bisexual, queer, polysexual, demisexual; (4) asexual or no sexuality; and (5) questioning, mostly heterosexual, or do not use labels.

Participants were asked what terms best describe their gender identity and were given a list: cisgender boy/man; transgender boy/man; cisgender girl/woman; transgender girl/woman; non-binary; genderqueer; genderfluid; pangender; agender; indigenous or other cultural gender minority identity; questioning or unsure of your gender identity; something else. This multiple-response measure was reduced to a single categorical variable for the current analyses: (1) cisgender, (2) transgender, (3) nonbinary, (4) agender, and (5) questioning or gender variant.

Race response options included White, Black or African American, Asian, Hawaiian or Pacific Islander, American Indian or Alaska Native, unknown or not reported, other, and prefer not to answer. Participants were able to mark all that applied to them. Participants were also asked if they were of Hispanic or Latino origin (yes/no/prefer not to answer).

Age was a continuous variable ranging from 13 to 22 years (M = 17.4 (2.2)). Sex assigned at birth was male, female, intersex, and prefer not to answer.

Type of community. Participants were asked to describe where they live, with the following response options: small town or rural area, suburban area next to a city, and urban or city area (and prefer not to answer).

### 2.3. Statistical Analyses

Missing data were less than <5% and were conservatively coded as “no” for dichotomous constructs and replaced with the mean for continuous constructs. This is based on procedures utilized across many national studies [50,51].

We applied latent class analysis to estimate profiles across self-reported SDOH deficits. To decide on the number of classes, we used the following fit statistics (summarized in Table 2): Akaike Information Criteria (AIC), Bayesian Information Criterion (BIC), Consistent Akaike Information Criterion (CAIC), and Consistent Akaike Information Criteria (CAIC), which are approximate fit indices where lower values indicate better fit. We also used likelihood-based tests—the Vuong–Lo–Mendell–Rubin adjusted likelihood ratio test (VLMR-LRT), which provides *p*-values assessing whether adding a class leads to a statistically significant improvement in model fit. We also considered how the selected models relate to each other (e.g., theoretically different), as well as the relative sizes of the classes. Here, we decided on a minimum profile size of 5% of the sample. Using the selected number of classes, we present class marginal means for each SDOH construct and plot these for graphic depiction.

We next report descriptive statistics for each SDOH deficit, average number of SDOH deficits, and health indicators for all participants as well as different sub-groups of youth based on different marginalized identities and/or contributors to health disparities (female assigned at birth, sexual minority identity, gender minority identity, Black race, American Indian or Alaska Native race, and living in a rural community). Finally, social identity and health indicators are statistically compared across classes using chi-square cross-tabulation (for categorical variables) of analysis of variance (for continuous variables). We conducted pairwise comparisons between the classes for each outcome.

## 3. Results

### 3.1. Classes of SDOH Deficits

**Figure 1:**
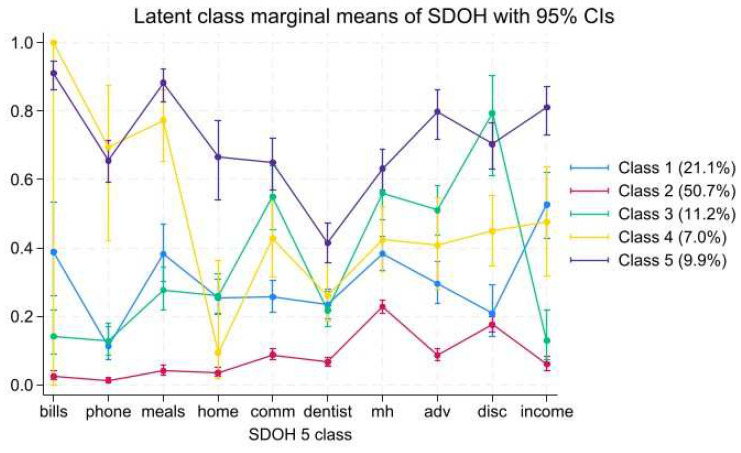
The selected 5-class solution. The measures used in the LCA are on the *x*-axis. Relative scores are presented on the *y*-axis.

Table 2 presents the fit statistics for two to six latent classes of SDOH deficits. Fit indices suggested a five-class solution. Marginal means for each SDOH deficit across classes are detailed in Table 3, and the classes are presented in Figure 1.

Class 1, “Economic Instability”, comprised 18.7% of the sample (n = 931) and had average probabilities across the different SDOH deficits with a slightly higher likelihood of economic instability.

Class 2, “Low Overall SDOH Deficits”, comprised 55.4% of the sample (n = 2760) and had the lowest probabilities across each SDOH deficit.

Class 3, “High Social SDOH Deficits”, comprised 9.1% of the sample (n = 454). This class was exemplified by high probabilities of social SDOH deficits (adversity and discrimination). This class also had the highest probabilities of barriers to mental health care and neighborhood disorder.

Class 4, “High Economic SDOH Deficits”, comprised 6.8% of the sample (n = 339) and was distinct in the high probabilities of economic instability, with more moderate probabilities of social, healthcare and neighborhood, and built environment deficits.

Class 5, “High Overall SDOH Deficits”, comprised 10.0% of the sample (n = 497) and was characterized by the highest probabilities for each SDOH deficit.

### 3.2. SDOH and Health Indicators by Different Marginalized Identities

Table 4 shows the distribution of SDOH deficits across the cohort overall and by marginalized identity/health disparity. Slightly over one-quarter (27.1%) of the cohort reported not having enough money to pay the bills, food insecurity (27.4%), low income (27.0%), high non-victimization adversity history (27.1%), and neighborhood disorder (25.5%). About one-third reported high discrimination history (32.3%) and barriers to mental health (35.2%). Less reported having their cell phone turned off (15.8%), going 2 or more years without seeing a dentist (16.6%), or home condition problems (17.3%). When examining cumulative deficits, the average number of SDOH deficits was 2.3 overall. The highest cumulative deficit average was seen among American Indian or Alaskan Natives (mean = 4.07, SD = 2.7). These individuals also reported the highest rates of most individual SDOH deficits, except those in the healthcare domain. Comparisons were not made across sub-groups due to an overlap in characteristics among participants.

Multiple mental and behavioral health indicators were also examined for the cohort overall and among different subgroups (Table 4). Overall, 28.4% of the cohort had tried to die by suicide at some point in their life, 78.5% reported lifetime thoughts of suicide (56.2% past year), 7.6% reported a drug overdose in their life, and 78.6% lifetime NSSI (65.4% past year). The average likelihood of living to age 35 was 75.6% for the sample (SD = 26.4), and the mean depression score was 28.9 (SD = 14.7). Recent alcohol use was reported by 19.5% of the cohort. Gender minority youth were among the most likely to report all of these health indicators (and lowest percent likelihood of living to age 35). Sexual minority youth and American Indian or Alaska Native youth also reported high levels of each health indicator.

### 3.3. Youth Social Identity Characteristics and Health Indicators Across Classes

Significant differences in social identity were noted across the classes (Table 5). Class 3, High Social SDOH Deficits, and Class 5, High Overall SDOH Deficits, were most likely to have participants who identified with a gender minority identity and a sexual minority identity. Class 4, High Economic SDOH deficits, and Class 5, High Overall SDOH Deficits, had the highest percentages of race and ethnic minority participants. More specifically, the High Economic SDOH Deficit class (4) had the highest percentage of Black youth, while the High Overall SDOH Deficit class (5) had the highest percentage of American Indian or Alaska Native youth; Hispanic or Latino youth were equally represented in both of these classes.

Health indicators varied significantly across classes (Table 5). Each class (1, 3, 4, and 5) had significantly higher proportions of each ealth indicator compared to Class 2, Low Overall SDOH Deficits. Class 3, High Social SDOH Deficits, and Class 5, High Overall SDOH Deficits, had the highest proportions of youth with each health indicator; these classes did not differ significantly from each other. For example, 50.2% of Class 3 reported a suicide attempt, as did 52.7% of Class 5. Class 4, High Economic SDOH Deficits, had a higher proportion of drug overdose and higher depression scores compared with Class 1, Economic Instability, and a lower proportion of suicidal ideation; Class 4 had a lower average likelihood of living to age 35—70.8% (vs. 75.9% in Class 1). Finally, when comparing Class 3, High Social SDOH Deficits, with Class 4, High Economic SDOH Deficits, Class 3 had significantly higher proportions of suicide attempts, suicidal ideation, NSSI, and depression, as well as a lower perceived likelihood of living to age 35.

## 4. Discussion

The current study applied a latent class analysis approach to explore patterns of self-reported SDOH deficits among a national U.S. sample of adolescents and young adults. Five different classes were identified, covering low, moderate, and high overall SDOH deficits, as well as a class that had notably higher proportions of economic instability and one with higher proportions of social SDOH deficits (adversity and discrimination). Classes were distinguished by both social identity and key health indicators—suicide, NSSI, drug overdose, depression, recent alcohol use, and the perceived likelihood of living to age 35. Findings offer important insights into our understanding of the intersection of SDOH, health indicators, marginalized identities, and health disparities.

Importantly, slightly more than half of the cohort was classified with low overall SDOH deficits, and participants in this class had the smallest proportions of all mental and behavioral health risk indicators, including suicide attempts and ideation, recent alcohol use, NSSI, drug overdose, and depression, as well as the highest likelihood of feeling they would live to age 35. This reinforces the idea that growing up in healthy communities, without economic struggles and with low adversity and discrimination, can help youth thrive. For example, a recent scoping review of community strengths found that markers like income and neighborhood characteristics, like access to green spaces, were important for mental health [52]. A study during COVID-19 found that access to play spaces, as well as social connections with friends, helped children’s well-being [53]. Community resources have been shown to be particularly important for youth with marginalized identities [54].

The remaining classes had higher proportions of young people who identified as members of groups who have documented health disparities based on sexuality, gender, race, and ethnicity were less likely to be members of the Low Overall SDOH Deficits group and overrepresented across the other four groups, all of which had higher rates of SDOH deficits. Sexual and gender minority participants were most likely to appear in the High Overall SDOH Deficits and the High Social SDOH Deficits groups, reflecting high rates of discrimination and non-victimization adversity. Youth who identified with a race that has historically been marginalized were most likely to be in groups that included high economic instability, consistent with research that shows that poverty is unequally distributed in the United States by race [55,56]. Indeed, Black youth were overrepresented in the High Economic SDOH Deficits class, which also includes neighborhood disorder. This class was high in health risks of drug overdose and depression. Poverty and income inequality have been linked to drug overdose rates [57,58]. Physical neighborhood disorder and poverty create high levels of stress, which may manifest in depression [59].

Mental and behavioral health indicators were also differentially associated with SDOH classes. All outcomes, suicide attempts, suicidal ideation, recent alcohol use, NSSI, depression, and likelihood of living to age 35 were the worst among the High Overall SDOH Deficits class. Further the High Social SDOH Deficits class did not differ on any of the health indicators from the High Overall SDOH Deficits class. Notably, these two classes had the highest proportions of sexual and gender minority participants, which is consistent with research on Minority Stress Theory that posits that unique stressors tied to discrimination and the victimization and violence that can co-occur add significant adversity burden to youth who identify as LGBTQ+ [15]. This distal burden in turn effects proximal views of self and variables like sense of mattering and belonging and can tax coping resources, leading to higher rates of mental health problems including depression, suicide, foreshortened views of the future, and substance use [11,16,17,18,19].

Instead of using demographic variables as proxies for risk factors, this study examines potentially modifiable contextual variables that affect adolescent and young adult health and that may explain mental and behavioral health disparities. The findings are consistent with previous work that highlights the importance of marginalized identities on risk for key mental and behavioral health indicators but puts these in connection with SDOH deficits [17,32]. It extends our knowledge by highlighting how different combinations of SDOH deficits across multiple sectors, including economic instability, social (adversity and discrimination) deficits, barriers to healthcare, and neighborhood and built environment have different impacts on health outcomes. This is consistent with growing research in the field about links between discrimination and health [33] and suicidal behaviors in particular [34].

### 4.1. Public Health Implications

The findings have important implications for healthcare practice and future research. Findings support broad public health approaches that recognize that true disease prevention is connected to policies that reduce poverty, improve built environments, and increase access to basic goods and services, including healthcare. These are modifiable, structural supports that can promote well-being and should be considered a cornerstone of prevention and mental health promotion. Too often we focus only on individual solutions such as access to mental health treatment. Such access to quality care is important but will likely not be fully effective without these larger policy and resource allocation strategies. The current study demonstrates the toll that high SDOH deficits take not only on mental and behavioral health symptoms but also on young people’s sense of their future. It is also important that the highest levels of mental health concerns were in the two groups (3 and 5) that had high levels of adversity and discrimination. This argues for using trauma-informed strategies as part of services for specific symptoms like substance use and depression. Further, policies and resources like gender affirming healthcare, as well as those that work against bullying and discrimination in schools and workplaces, may help create climates that work against the corrosive effects of minority stress [60].

### 4.2. Limitations

Although recruitment advertisements encouraged youth to ‘have their voice heard’ and ‘make a difference’ to help reduce self-selection bias based upon interest in the survey topic, the opt-in nature of this methodology means the results should not be considered representative of young people in this age group across the U.S. Data are self-reported and are thus susceptible to under- or over-reporting of responses. The cross-sectional nature of these analyses limits inferences about temporal associations. The class entropy value, which indicates how well the model does at assigning individuals to specific classes, was lower than 0.80. Additional SDOH deficit indicators might improve the ability to assign participants to classes. This study only included some of the many key public health indicators. Future research should continue to explore these research questions among a broader range of health outcomes.

## 5. Conclusions

Concerns about adolescent and young adult mental and behavioral health have been building over the last decade as rates of youth suicide and drug overdose fatalities have increased despite significant work trying to decrease these deaths. The findings from this study support the significant impact of social and environmental factors on these health indicators. A public health approach that recognizes that improving the health of today’s young people must be connected to policies that reduce poverty, discrimination, and adversity; improve neighborhoods; and increase access to basic goods, services, and healthcare is critical. This is particularly salient for marginalized groups like sexual and gender minority and racial and ethnic minority youth.

## Figures and Tables

**Table 1:** Demographic characteristics (N = 4981).

Characteristic	n	%
Age		
13–17 years	2835	56.9
18–22 years	2146	43.1
Mean age (SD)	---	17.4 (2.2)
Sex at birth		
Male	1868	37.5
Female	2931	58.8
Intersex	46	0.9
Missing	136	2.7
Gender identity ^a^		
Exclusively cisgender	3074	61.7
Transgender	751	15.1
Non-binary, genderqueer, genderfluid, pangender, indigenous	1206	24.2
Agender	204	4.1
Gender variant, questioning	348	7.0
Missing	85	1.7
Sexual identity ^a^		
Exclusively heterosexual	2005	40.3
Gay, lesbian	674	13.5
Bisexual, queer, polysexual, demisexual	1927	38.7
Asexual, no sexuality	406	8.1
Questioning, mostly heterosexual, no labels	249	5.0
Missing	64	1.3
Race ^a^		
White	3711	74.5
Black or African American	557	11.2
Asian	572	11.5
Hawaiian or Pacific Islander	56	1.1
American Indian or Alaska Native	217	4.4
Missing	394	7.9
Hispanic or Latino origin (any race)		
No	3858	77.5
Yes	1071	21.5
Missing	52	1.0
Type of community		
Small town or rural area	1470	29.5
Suburban area next to a city	2462	49.4
Urban or city area	958	19.2
Missing	91	1.8
Family income		
Lower than the average family	1346	27.0
About the same as the average family	2279	45.7
Higher than the average family	1174	23.6
Missing	182	3.7

^a^ Multiple responses possible.

**Table 2:** Fit statistics for varying size latent class models of SDOH.

Class	Log Likelihood	AIC	BIC	AICc	CAIC	Entropy	VLMR	*p* > VLMR
1	−27,6225.67	55,265.34	55,330.48	55,265.39	55,340.48	---	---	---
2	−25,095.98	50,233.96	50,370.74	50,234.15	50,391.74	0.78	5053.38	<0.001
3	−24,863.51	49,791.02	49,999.45	49,791.45	50,031.45	0.64	464.94	<0.001
4	−24,722.11	49,530.22	49,810.30	49,530.99	49,853.30	0.66	282.80	<0.001
**5**	**−24,686.24**	**49,480.48**	**49,832.20**	**49,481.68**	**49,886.20**	**0.70**	**71.75**	**<0.001**
6	−24,665.82	49,461.63	49,885.00	49,463.38	49,950.00	0.62	40.85	0.16

Note. AIC = Akaike Information Criterion; BIC = Bayesian Information Criterion; AICc = Corrected Akaike Information Criterion, CACI = Consistent Akaike Information Criterion, VLMR = Vuong–Lo–Mendall–Rubin test. Bold values reflect the best fit using these indices.

**Table 3:** Latent class marginal means.

Characteristic	Class 1 Economic Instability (n = 931)	Class 2 Low Overall SDOH Deficits (n = 2760)	Class 3 High Social SDOH Deficits (n = 454)	Class 4 High Economic SDOH Deficits (n = 339)	Class 5 High Overall SDOH Deficits (n = 497)
	Margin (SE)	Margin (SE)	Margin (SE)	Margin (SE)	Margin (SE)
Type of SDOH deficit					
Economic instability					
Not enough money to pay the bills	0.39 (0.07)	0.02 (0.2)	0.14 (0.03)	1.00 (0.001)	0.91 (0.02)
Cell phone turned off	0.11 (0.02)	0.01 (0.00)	0.13 (0.02)	0.65 (0.12)	0.65 (0.03)
Food insecurity	0.38 (0.04)	0.04 (0.01)	0.28 (0.03)	0.88 (0.05)	0.88 (0.02)
Low income	0.53 (0.05)	0.06 (0.01)	0.13 (0.04)	0.47 (0.08)	0.81 (0.03)
Social context					
Non-victimization adversity	0.29 (0.03)	0.09 (0.01)	0.51 (0.04)	0.41 (0.07)	0.80 (0.04)
Discrimination	0.21 (0.04)	0.17 (0.01)	0.79 (0.07)	0.45 (0.05)	0.70 (0.03)
Healthcare					
Barriers to mental health care	0.38 (0.03)	0.23 (0.01)	0.56 (0.04)	0.42 (0.05)	0.63 (0.03)
2+ years since seeing dentist	0.23 (0.02)	0.07 (0.01)	0.22 (0.03)	0.26 (0.04)	0.41 (0.03)
Neighborhood and built environment					
Home condition problems	0.25 (0.03)	0.03 (0.01)	0.26 (0.03)	0.09 (0.07)	0.67 (0.06)
Neighborhood disorder	0.26 (0.02)	0.09 (0.01)	0.55 (0.05)	0.43 (0.06)	0.65 (0.04)

**Table 4:** Individual social and structural determinants of health deficits and health indicators for different groups of young people.

	All Participants (N = 4981)	Female Assigned at Birth (n = 2931)	Sexual Minority Identity (n = 2909)	Gender Minority Identity (n = 1814)	Black Race (n = 557)	American Indian or Alaska Native Race (n = 217)	Lives in Rural Area (n = 1470)
	n (%)	n (%)	n (%)	n (%)	n (%)	n (%)	n (%)
Type of SDOH							
Economic instability							
Not enough money to pay the bills	1349 (27.1)	839 (28.6)	850 (29.2)	559 (30.8)	208 (37.3)	99 (45.6)	444 (30.2)
Cell phone turned off	788 (15.8)	475 (15.2)	494 (17.0)	335 (18.5)	168 (30.2)	72 (33.2)	283 (19.3)
Food insecurity	1366 (27.4)	833 (28.4)	898 (30.9)	596 (32.9)	218 (39.1)	99 (45.6)	434 (29.5)
Low income	1346 (27.0)	795 (27.1)	859 (29.5)	561 (30.9)	191 (34.3)	91 (41.9)	443 (30.1)
Social context							
Non-victimization adversity	1349 (27.1)	827 (28.2)	953 (32.8)	669 (36.9)	166 (29.8)	113 (52.1)	454 (30.9)
Discrimination	1610 (32.3)	941 (32.1)	1132 (38.9)	843 (46.5)	200 (35.9)	118 (54.4)	526 (35.8)
Healthcare							
Barriers to mental health care	1753 (35.2)	1132 (38.6)	1204 (41.4)	824 (45.4)	210 (37.7)	86 (39.6)	527 (35.9)
2+ years since seeing dentist	829 (16.6)	470 (16.0)	533 (18.3)	368 (20.3)	131 (23.5)	48 (22.1)	253 (17.2)
Neighborhood and built environment							
Home condition problems	864 (17.3)	544 (18.6)	592 (20.3)	401 (22.1)	102 (18.3)	63 (29.0)	318 (21.6)
Neighborhood disorder	1269 (25.5)	786 (26.8)	843 (29.0)	571 (31.5)	184 (33.0)	95 (43.8)	334 (22.7)
Average number of SDOH deficits	2.51 (2.3)	2.61 (2.4)	2.87 (2.4)	3.16 (2.5)	3.19 (2.4)	4.07 (2.7)	2.73 (2.4)
Health indicators							
Suicide attempt (lt)	1413 (28.4)	904 (30.8)	1085 (37.3)	806 (44.4)	153 (27.5)	97 (44.7)	431 (29.3)
Thoughts of suicide (lt)	3911 (78.5)	2366 (80.7)	2555 (87.8)	1669 (92.0)	413 (74.1)	180 (82.9)	1160 (78.9)
Thoughts of suicide (py)	2799 (56.2)	1740 (59.4)	1845 (63.4)	1227 (67.6)	266 (47.8)	142 (65.4)	847 (57.6)
Drug overdose (lt)	378 (7.6)	267 (9.1)	308 (10.6)	229 (12.6)	40 (7.2)	31 (14.3)	119 (8.1)
Alcohol use (past 2 weeks)	974 (19.5)	537 (18.3)	627 (21.5)	363 (20.0)	74 (13.3)	42 (19.3)	280 (19.1)
NSSI (lt)	3914 (78.6)	2409 (82.2)	2553 (87.8)	1657 (91.3)	414 (74.3)	183 (84.3)	1177 (80.1)
NSSI (py)	3259 (65.4)	2031 (69.3)	2204 (75.8)	1473 (81.2)	340 (61.0)	160 (73.7)	1006 (68.4)
Likelihood of living to age 35 (% (SD))	75.6 (26.4)	74.5 (26.9)	69.1 (27.7)	63.5 (28.7)	77.2 (24.2)	63.6 (30.1)	72.7 (27.7)
Depression (M (SD))	28.9 (14.7)	31.0 (14.6)	33.0 (14.1)	35.6 (13.9)	28.5 (14.5)	34.7 (15.9)	30.1 (14.7)

SDOH = social determinants of health. lt = lifetime; py = past year.

**Table 5:** Youth characteristics across classes.

Characteristic	Class 1 Economic Instability n (%)	Class 2 Low Overall SDOH Deficits n (%)	Class 3 High Social SDOH Deficits n (%)	Class 4 High Economic SDOH Deficits n (%)	Class 5 High Overall SDOH Deficits n (%)	*p* Value
Identity						
Mean age	17.95	17.26 ^a^	17.01 ^a^	17.58 ^c^	17.76 ^a,b,c^	<0.001
Age category						
13–17 years	446 (47.9)	1662 (60.2) ^a^	289 (63.7) ^a^	189 (55.7) ^a,c^	249 (50.1) ^b,c^	<0.001
18–22 years	485 (52.1)	1098 (39.8)	165 (36.3)	150 (44.3)	248 (49.9)	
Sex assigned at birth						
Male	354 (38.8)	1123 (41.5)	138 (32.2) ^a,b^	110 (34.2) ^a,b^	143 (30.2) ^a,b^	<0.001
Female	556 (60.9)	1573 (58.1)	280 (65.3)	206 (64.0)	316 (66.7)	
Intersex	3 (0.3)	11 (0.4)	11 (2.6)	6 (1.9)	15 (3.2)	
Gender identity						
Cisgender	602 (66.7)	1861 (69.6) ^a^	186 (42.9) ^a,b^	192 (60.6) ^b,c^	212 (44.4) ^a,b,d^	<0.001
Transgender	61 (6.8)	221 (8.3)	62 (14.3)	29 (9.1)	69 (14.5)	
Nonbinary	153 (17.0)	352 (13.2)	107 (24.7)	62 (19.6)	123 (25.8)	
Agender	29 (3.2)	83 (3.1)	22 (5.1)	9 (2.8)	27 (5.7)	
Questioning	57 (6.3)	156 (5.8)	57 (13.1)	25 (7.9)	46 (9.6)	
Any gender minority						
No	623 (66.9)	1911 (69.3)	199 (43.8) ^a,b^	209 (61.7) ^b,c^	224 (45.1) ^a,b,d^	<0.001
Yes	308 (33.1)	848 (30.7)	255 (56.2)	130 (38.3)	273 (54.9)	
Sexual identity						
Exclusively heterosexual	375 (41.5)	1260 (46.6) ^a^	103 (23.4) ^a,b^	129 (39.2) ^b,c^	124 (25.9) ^a,b,d^	<0.001
Gay or lesbian	102 (11.3)	298 (11.0)	76 (17.3)	33 (10.0)	69 (14.4)	
Bisexual	311 (34.4)	799 (29.5)	197 (44.8)	128 (38.9)	227 (47.5)	
Asexual	69 (7.6)	184 (6.8)	25 (5.7)	18 (5.5)	33 (6.9)	
Questioning	47 (5.2)	164 (6.1)	39 (8.9)	21 (6.4)	25 (5.2)	
Any sexual minority						
No	396 (42.5)	1295 (46.9) ^a^	112 (24.7) ^a,b^	138 (40.7) ^b,c^	131 (26.4) ^a,b,d^	<0.001
Yes	535 (57.5)	1465 (53.1)	342 (75.3)	201 (59.3)	366 (73.6)	
Race						
White	695 (74.7)	2098 (76.0)	338 (74.5)	216 (63.7) ^a,b,c^	364 (73.2) ^d^	<0.001
Black	121 (13.0)	227 (8.2) ^a^	52 (11.5) ^b^	74 (21.8) ^a,b,c^	83 (16.7) ^b,c^	<0.001
Asian	89 (9.6)	385 (13.9) ^a^	47 (10.3) ^b^	22 (6.5) ^b^	29 (5.8) ^a,b,c^	<0.001
American Indian or Alaska Native	45 (4.8)	64 (2.3) ^a^	28 (6.2) ^b^	25 (7.4) ^b^	55 (11.1) ^a,b,c^	<0.001
Any race minority						
No	686 (73.7)	2094 (75.9)	333 (73.3)	228 (67.3) ^a,b^	342 (68.8) ^a,b^	<0.001
Yes	245 (26.3)	666 (24.1)	121 (26.7)	111 (32.7)	155 (31.2)	
Hispanic or Latino						
No	683 (73.4)	2259 (81.9) ^a^	348 (76.7) ^b^	254 (74.9) ^b^	366 (73.6) ^b^	<0.001
Yes	248 (26.6)	501 (18.1)	106 (23.3)	85 (25.1)	131 (26.4)	
Type of community						
Rural	298 (32.5)	744 (27.2) ^a^	136 (30.5) ^b^	116 (37.4) ^b,c^	176 (36.3) ^b,c^	<0.001
Suburban	393 (42.9)	1560 (57.1)	220 (49.3)	109 (35.2)	180 (37.1)	
Urban	226 (24.7)	428 (15.7)	90 (20.2)	85 (27.4)	129 (26.6)	
Health indicators						
Suicide attempt (lt)	272 (29.2)	545 (19.7) ^a^	228 (50.2) ^a,b^	106 (31.3) ^b,c^	262 (52.7) ^a,b,d^	<0.001
Thoughts of suicide (lt)	766 (82.3)	2014 (73.0) ^a^	427 (94.1) ^a,b^	251 (74.0) ^a,c^	453 (91.1) ^a,b,d^	<0.001
Thoughts of suicide (py)	527 (56.6)	1408 (51.0) ^a^	324 (71.4) ^a,b^	187 (55.2) ^c^	353 (71.0) ^a,b,d^	<0.001
Drug overdose (lt)	61 (6.55)	125 (4.5) ^a^	66 (14.5) ^a,b^	35 (10.3) ^a,b^	91 (18.3) ^a,b,d^	<0.001
Alcohol use (past 2 weeks)	200 (21.5)	470 (17.0) ^a^	91 (20.0)	69 (20.3)	144 (29.0) ^a,b,c,d^	<0.001
NSSI (lt)	758 (81.4)	2027 (73.4) ^a^	417 (91.9) ^a,b^	261 (77.0) ^c^	451 (90.7) ^a,b,d^	<0.001
NSSI (py)	620 (66.6)	1635 (59.2) ^a^	374 (82.4) ^a,b^	220 (64.9) ^b,c^	410 (82.5) ^a,b,d^	<0.001
Likelihood of living to age 35 (%)	75.95	81.46 ^a^	62.09 ^a,b^	70.77 ^a,b,c^	58.45 ^a,b,d^	<0.001
Depression (M)	28.76	24.55 ^a^	39.80 ^a,b^	32.24 ^a,b,c^	41.76 ^a,b,d^	<0.001

^a^ significantly from Class 1; ^b^ significantly from Class 2; ^c^ significantly from Class 3; ^d^ significantly from Class 4.

## Data Availability

The data that support the findings of this study are available from the corresponding author upon reasonable request.

## Supplementary Materials

The following supporting information can be downloaded at: https://www.mdpi.com/article/10.3390/ijerph22071013/s1, Table S1: Original and transformed SDOH items; Table S2: Correlations between different social and structural determinants of health deficits.

## References

[1] Daly M. (2022). Prevalence of depression among adolescents in the US from 2009 to 2019: Analysis of trends by sex, race/ethnicity, and income. J. Adolesc. Health.

[2] Xiao Q., Song X., Huang L., Hou D., Huang X. (2022). Global prevalence and characteristics of non-suicidal self-injury between 2010 and 2021 among a non-clinical sample of adolescents: A meta-analysis. Front. Psychiatry.

[3] De Luca L., Pastore M., Palladino B.E., Reime B., Warth P., Menesini E. (2023). The development of Non-Suicidal Self-Injury (NSSI) during adolescence: A systematic review and Bayesian meta-analysis. J. Affect. Disord..

[4] Moloney F., Amini J., Sinyor M., Schaffer A., Lanctôt K.L., Mitchell R.H. (2024). Sex differences in the global prevalence of nonsuicidal self-injury in adolescents: A meta-analysis. JAMA Netw. Open.

[5] Borowsky I.W., Ireland M., Resnick M.D. (2009). Health status and behavioral outcomes for youth who anticipate a high likelihood of early death. Pediatrics.

[6] Xiao Y., Lu W. (2019). Cumulative health risk behaviors and adolescent suicide: The moderating role of future orientation. Am. J. Health Behav..

[7] Wang Y.-J., Li X., Ng C.H., Xu D.-W., Hu S., Yuan T.-F. (2022). Risk factors for non-suicidal self-injury (NSSI) in adolescents: A meta-analysis. EClinicalMedicine.

[8] Grossberg A., Rice T. (2023). Depression and suicidal behavior in adolescents. Med. Clin..

[9] Lyons R.M., Yule A.M., Schiff D., Bagley S.M., Wilens T.E. (2019). Risk factors for drug overdose in young people: A systematic review of the literature. J. Child Adolesc. Psychopharmacol..

[10] Oshin L., Hausmann-Stabile C., Meza J.I., Polanco-Roman L., Bettis A.H., Reyes-Portillo J., Benton T.D. (2022). Suicide and suicidal behaviors among minoritized youth. Child Adolesc. Psychiatr. Clin..

[11] Baiden P., LaBrenz C.A., Asiedua-Baiden G., Muehlenkamp J.J. (2020). Examining the intersection of race/ethnicity and sexual orientation on suicidal ideation and suicide attempt among adolescents: Findings from the 2017 Youth Risk Behavior Survey. J. Psychiatr. Res..

[12] Friedman J., Beletsky L., Jordan A. (2022). Surging racial disparities in the US overdose crisis. Am. J. Psychiatry.

[13] Brinzo P.N., Martins S.S. (2024). Racial/ethnic trends in opioid and polysubstance opioid overdose mortality in adolescents and young adults, 1999–2020. Addict. Behav..

[14] Friedman J., Godvin M., Shover C.L., Gone J.P., Hansen H., Schriger D.L. (2022). Trends in drug overdose deaths among US adolescents, January 2010 to June 2021. JAMA.

[15] Meyer I.H. (2003). Prejudice, social stress, and mental health in lesbian, gay, and bisexual populations: Conceptual issues and research evidence. Psychol. Bull..

[16] Burke T.A., Bettis A.H., Barnicle S.C., Wang S.B., Fox K.R. (2021). Disclosure of self-injurious thoughts and behaviors across sexual and gender identities. Pediatrics.

[17] Mereish E.H., Peters J.R., Brick L.A., Killam M.A., Yen S. (2023). A daily diary study of minority stressors, suicidal ideation, nonsuicidal self-injury ideation, and affective mechanisms among sexual and gender minority youth. J. Psychopathol. Clin. Sci..

[18] Dennis M.T., Davis C.G. (2025). Out of sight but on my mind: Distal stressors, identity concealment, proximal stress, and mental health among sexual or gender minorities. Sex. Res. Soc. Policy.

[19] Testa R.J., Michaels M.S., Bliss W., Rogers M.L., Balsam K.F., Joiner T. (2017). Suicidal ideation in transgender people: Gender minority stress and interpersonal theory factors. J. Abnorm. Psychol..

[20] Klonsky E.D., Pachkowski M.C., Shahnaz A., May A.M. (2021). The three-step theory of suicide: Description, evidence, and some useful points of clarification. Prev. Med..

[21] Kaniuka A.R., Nanney E.M., Robertson R., Hoff R., Smith M., Bowling J., Basinger E.D., Dahl A.A., Cramer R.J. (2024). A grounded theory of sexual and gender minority suicide risk: The sexual and gender minority suicide risk and protection model. Psychol. Sex. Orientat. Gend. Divers..

[22] Goldbach J.T., Tanner-Smith E.E., Bagwell M., Dunlap S. (2014). Minority stress and substance use in sexual minority adolescents: A meta-analysis. Prev. Sci..

[23] Parent M.C., Arriaga A.S., Gobble T., Wille L. (2019). Stress and substance use among sexual and gender minority individuals across the lifespan. Neurobiol. Stress.

[24] Hunter J., Butler C., Cooper K. (2021). Gender minority stress in trans and gender diverse adolescents and young people. Clin. Child Psychol. Psychiatry.

[25] Ahrenholtz M.S., Nicholas J., Sacco A., Bresin K. (2025). Sexual and gender minority stress in nonsuicidal self-injury engagement: A meta-analytic review. Suicide Life-Threat. Behav..

[26] WHO Commission on Social Determinants of Health (2008). Closing the Gap in a Generation: Health Equity Through Action on the Social Determinants of Health: Commission on Social Determinants of Health Final Report.

[27] Solar O., Irwin A. (2010). A Conceptual Framework for Action on the Social Determinants of Health.

[28] Frank J., Abel T., Campostrini S., Cook S., Lin V.K., McQueen D.V. (2020). The social determinants of health: Time to re-think?. Int. J. Environ. Res. Public Health.

[29] World Health Organization Social Determinants of Health. https://www.who.int/health-topics/social-determinants-of-health#tab=tab_1.

[30] Hatzenbuehler M.L., Pachankis J.E. (2016). Stigma and minority stress as social determinants of health among lesbian, gay, bisexual, and transgender youth: Research evidence and clinical implications. Pediatr. Clin..

[31] Gallagher K., Phillips G., Corcoran P., Platt S., McClelland H., O’Driscoll M., Griffin E. (2025). The social determinants of suicide: An umbrella review. Epidemiol. Rev..

[32] Hill-Briggs F., Adler N.E., Berkowitz S.A., Chin M.H., Gary-Webb T.L., Navas-Acien A., Thornton P.L., Haire-Joshu D. (2020). Social determinants of health and diabetes: A scientific review. Diabetes Care.

[33] Kammer-Kerwick M., Cox K., Purohit I., Watkins S.C. (2024). The role of social determinants of health in mental health: An examination of the moderating effects of race, ethnicity, and gender on depression through the all of us research program dataset. PLOS Ment. Health.

[34] Henderson E.R., Goldbach J.T., Blosnich J.R. (2022). Social determinants of sexual and gender minority mental health. Curr. Treat. Options Psychiatry.

[35] Monroe P., Campbell J.A., Harris M., Egede L.E. (2023). Racial/ethnic differences in social determinants of health and health outcomes among adolescents and youth ages 10–24 years old: A scoping review. BMC Public Health.

[36] Wang G., Wu L. (2021). Social determinants on suicidal thoughts among young adults. Int. J. Environ. Res. Public Health.

[37] Na P.J., Shin J., Kwak H.R., Lee J., Jester D.J., Bandara P., Kim J.Y., Moutier C.Y., Pietrzak R.H., Oquendo M.A. (2025). Social determinants of health and suicide-related outcomes: A review of meta-analyses. JAMA Psychiatry.

[38] Vince R.A., Jiang R., Bank M., Quarles J., Patel M., Sun Y., Hartman H., Zaorsky N.G., Jia A., Shoag J. (2023). Evaluation of social determinants of health and prostate cancer outcomes among black and white patients: A systematic review and meta-analysis. JAMA Netw. Open.

[39] Tran R., Forman R., Mossialos E., Nasir K., Kulkarni A. (2022). Social determinants of disparities in mortality outcomes in congenital heart disease: A systematic review and meta-analysis. Front. Cardiovasc. Med..

[40] Wilder M.E., Kulie P., Jensen C., Levett P., Blanchard J., Dominguez L.W., Portela M., Srivastava A., Li Y., McCarthy M.L. (2021). The impact of social determinants of health on medication adherence: A systematic review and meta-analysis. J. Gen. Intern. Med..

[41] Kangas T., Milis S.-L., Vanthomme K., Vandenheede H. (2025). The social determinants of health-related quality of life among people with chronic disease: A systematic literature review. Qual. Life Res..

[42] American Psychological Association DSM-5 Self-Rated Level 1 Cross-Cutting Symptom Measure-Child Age 11–17. https://www.psychiatry.org/psychiatrists/practice/dsm/educational-resources/assessment-measures.

[43] National Data Archive Non-Suicidal Self-Injury. National Insttute of Mental Health. https://nda.nih.gov/.

[44] Turner H.A., Butler M.J. (2003). Direct and indirect effects of childhood adversity on depressive symptoms in young adults. J. Youth Adolesc..

[45] Ebesutani C., Reise S.P., Chorpita B.F., Ale C., Regan J., Young J., Higa-McMillan C., Weisz J.R. (2012). The Revised Child Anxiety and Depression Scale-Short Version: Scale reduction via exploratory bifactor modeling of the broad anxiety factor. Psychol. Assess..

[46] Klaufus L., Verlinden E., Van Der Wal M., Kösters M., Cuijpers P., Chinapaw M. (2020). Psychometric evaluation of two short versions of the Revised Child Anxiety and Depression Scale. BMC Psychiatry.

[47] American Academy of Family Physicians Social Needs Screening Tool. https://www.aafp.org/dam/AAFP/documents/patient_care/everyone_project/hops19-physician-form-sdoh.pdf.

[48] Williams D.R., Yu Y., Jackson J.S., Anderson N.B. (1997). Racial differences in physical and mental health: Socio-economic status, stress and discrimination. J. Health Psychol..

[49] Perkins D.D., Florin P., Rich R.C., Wandersman A., Chavis D.M. (1990). Participation and the social and physical environment of residential blocks: Crime and community context. Am. J. Community Psychol..

[50] Ybarra M.L., Espelage D.L., Mitchell K.J. (2007). The co-occurrence of Internet harassment and unwanted sexual solicitation victimization and perpetration: Associations with psychosocial indicators. J. Adolesc. Health.

[51] Banyard V., Mitchell K.J., Ybarra M.L. (2021). Exposure to self-directed violence: Understanding intention to help and helping behaviors among adolescents and emerging adults. Int. J. Environ. Res. Public Health.

[52] Banyard V., Rousseau D., Shockley McCarthy K., Stavola J., Xu Y., Hamby S. (2025). Community-level characteristics associated with resilience after adversity: A scoping review of research in urban locales. Trauma Violence Abus..

[53] Mitra R., Waygood E.O.D., Fullan J. (2021). Subjective well-being of Canadian children and youth during the COVID-19 pandemic: The role of the social and physical environment and healthy movement behaviours. Prev. Med. Rep..

[54] Ceatha N., Koay A.C., Buggy C., James O., Tully L., Bustillo M., Crowley D. (2021). Protective factors for LGBTI+ youth wellbeing: A scoping review underpinned by recognition theory. Int. J. Environ. Res. Public Health.

[55] Heard-Garris N., Boyd R., Kan K., Perez-Cardona L., Heard N.J., Johnson T.J. (2021). Structuring poverty: How racism shapes child poverty and child and adolescent health. Acad. Pediatr..

[56] Baker R.S., Brady D., Parolin Z., Williams D.T. (2022). The enduring significance of ethno-racial inequalities in poverty in the US, 1993–2017. Popul. Res. Policy Rev..

[57] Fink D.S., Keyes K.M., Branas C., Cerdá M., Gruenwald P., Hasin D. (2023). Understanding the differential effect of local socio-economic conditions on the relation between prescription opioid supply and drug overdose deaths in US counties. Addiction.

[58] Fink D.S., Schleimer J.P., Keyes K.M., Branas C.C., Cerdá M., Gruenwald P., Hasin D. (2024). Social and economic determinants of drug overdose deaths: A systematic review of spatial relationships. Soc. Psychiatry Psychiatr. Epidemiol..

[59] Zimmerman A., Lund C., Araya R., Hessel P., Sanchez J., Garman E., Evans-Lacko S., Diaz Y., Avendano-Pabon M. (2022). The relationship between multidimensional poverty, income poverty and youth depressive symptoms: Cross-sectional evidence from Mexico, South Africa and Colombia. BMJ Glob. Health.

[60] Fish J.N., Dunkwu L., Tchangalova N., McFarlane S. (2025). Associations between policy and health for sexual and gender minority youth in the United States: A scoping review. J. Adolesc. Health.

