# Supplementary material for: The Contribution of Social and Structural Determinants of Health Deficits to Mental and Behavioral Health Among a Diverse Group of Young People
Source: Int J Environ Res Public Health. 2025 Jun 26;22(7):1013. doi: 10.3390/ijerph22071013 (PMC12294719; doi:10.3390/ijerph22071013)
Supplement: Supplementary file 1 [file ijerph-22-01013-s001.zip › Supplementary Table S1.pdf]

Supplementary Table S1. Original and transformed SDOH items

Type of SDOH	Original item wording	Original scale item M (SD)	Original scale item Range	Z-scored item Unweighted n (%)
Economic instability				
Not enough money to pay the bills	"How often did this describe you or your family in the past 12 months? We did not have enough money to pay the bills." (1 item)	0.84 (1.1)	0 - 4	1,349 (27.1)
Cell phone turned off	"In the past 12 months, how often has your cell phone been turned off because you or your family did not have enough money to pay the bill." (1 item)	0.24 (0.7)	0 – 4	788 (15.8)
Food insecurity	"In the past 30 days, how often did you skip meals or eat less because you or your family didn't have enough money for food?" (1 item)	0.49 (0.9)	0 – 4	1,366 (27.4)
Social context				
Non-victimization adversity	"Now I'd like to ask you about some other experiences that you or someone in your family may have had" (11 items)	3.20 (2.3)	0 – 11	1,349 (27.1)
Discrimination	"In your day-to-day life, how often did any of the following things happen to you?" (9 items)	5.67 (2.8)	0 – 9	1,610 (32.3)
Healthcare				
Last time saw dentist	"When was the last time you saw a dentist for a check-up, exam, teeth cleaning, or other dental work?" (1 item)			
	More than 2 years ago / never	---	---	829 (16.6)
Barriers to mental health care	"Please rate each of the possible concerns that might affect your decision to seek treatment for a mental health problem from a professional, like a psychologist or counselor." (5 items)	2.99 (1.0)	1 – 5	1,753 (35.2)
Neighborhood and built environment				

Type of SDOH	Original item wording	Original scale item		Z-scored item
		M (SD)	Range	Unweighted n (%)
Home condition problems	"Now, please think about where you **live now.** Are there any of these problems where you live? Please think about your permanent place of residence, not a dorm room or other temporary housing.*" (8 items)*	0.65 (1.1)	0 – 8	864 (17.3)
Neighborhood disorder	"Please tell me if each of the following was a serious problem, minor problem, or no problem at all in your neighborhood... by neighborhood we mean the street you lived on and a few streets around it." (12 items)	0.40 (0.4)	0 - 2	1,269 (25.5)

SDOH = social determinants of health
