# Supplementary material for: The Contribution of Social and Structural Determinants of Health Deficits to Mental and Behavioral Health Among a Diverse Group of Young People
Source: Int J Environ Res Public Health. 2025 Jun 26;22(7):1013. doi: 10.3390/ijerph22071013 (PMC12294719; doi:10.3390/ijerph22071013)
Supplement: Supplementary file 1 [file ijerph-22-01013-s001.zip › Supplementary Table S2.pdf]

Supplementary Table S2. Correlations between different social and structural determinants of health deficits

	**1**	**2**	**3**	**4**	**5**	**6**	**7**	**8**	**9**	**10**
Not enough money to pay the bills (1)	1.0	---	---	---	---	---	---	---	---	---
Cell phone turned off (2)	.48	1.0	---	---	---	---	---	---	---	---
Food insecurity (3)	.49	.41	1.0	---	---	---	---	---	---	---
Low income (4)	.42	.27	.35	1.0						
Non-victimization adversity (5)	.31	.27	.30	.25	1.0	---	---	---	---	---
Discrimination (6)	.20	.19	.21	.11	.25	1.0	---	---	---	---
Barriers to mental health care (7)	.16	.12	.17	.13	.12	.18	1.0	---	---	---
2+ years since seeing dentist (8)	.19	.18	.18	.18	.18	.08	.09	1.0	---	---
Home condition problems (9)	.25	.23	.28	.27	.22	.17	.17	.15	1.0	---
Neighborhood problems (10)	.25	.26	.26	.18	.24	.23	.16	.12	.23	1.0

Note. All pairwise correlations significant at $p \leq .001$.
